# Direct Public-Payer Costs of Dialysis Therapies in Greece: A Patient-Level Micro-Costing Study Across Modality and Provider Sector

**DOI:** 10.3390/jmahp14030044

**Published:** 2026-07-28

**Authors:** Vasileios Zavvos, John Fanourgiakis, Michael A. Talias, Aimilia Zachariou, Marios Papasotiriou, Christos Iatrou, Nikolaos Kontodimopoulos, Christos Ntais

**Affiliations:** 1 Healthcare Management Program, School of Social Sciences, Hellenic Open University, 26335 Patras, Greece; 2 Centre for Nephrology and Dialysis Unit, HYGEIA Hospital, 15123 Athens, Greece; 3 Department of Management Science and Technology, Hellenic Mediterranean University, 72100 Agios Nikolaos, Greece; jfanourgiakis@hmu.gr; 4 Healthcare Management Program, School of Economics & Management, Open University of Cyprus, Nicosia 2220, Cyprus; 5 Protypo Dialysis Centre of Piraeus, 18233 Piraeus, Greece; 6 Department of Nephrology and Kidney Transplantation, School of Medicine, University of Patras, 26504 Rio, Greece; 7 Department of Economics and Sustainable Development, Harokopio University, 17676 Athens, Greece

**Keywords:** hemodialysis, peritoneal dialysis, micro-costing, public payer, health expenditure, Greece

## Abstract

Background: Dialysis is a life-sustaining but resource-intensive treatment for patients with kidney failure. Contemporary Greek evidence on the public-payer cost of dialysis is limited, particularly for comparisons across modality, provider sector and treatment subtype. Methods: We performed a retrospective observational micro-costing study using 2022 patient-level data from five hemodialysis units and one peritoneal dialysis unit in Greece. The perspective was that of the public payer and public health budget. Annual costs included dialysis treatment and consumables, patient allowances and transport reimbursement, dialysis-related drugs, laboratory and imaging tests, hospitalizations and public dialysis-unit operating costs where applicable. Results: The analytic sample included 359 patients: 337 receiving hemodialysis and 22 receiving peritoneal dialysis. Median annual total cost was EUR 35,902.53 for hemodialysis and EUR 57,136.44 for peritoneal dialysis per patient. Peritoneal dialysis generated higher annual cost than hemodialysis, mainly because of treatment and consumable costs, and remained more costly after excluding dialysis-unit operating costs in sensitivity analysis. Public-sector hemodialysis had higher recorded median annual public-payer/public-budget cost than private-sector hemodialysis (EUR 38,794.55 versus EUR 34,836.98), but this difference was no longer statistically significant after excluding dialysis-unit operating costs. Hemodiafiltration was more costly than conventional hemodialysis, and automated peritoneal dialysis was more costly than continuous ambulatory peritoneal dialysis. National extrapolation estimated annual public expenditure of EUR 466.6 million using mean costs and EUR 446.8 million using median-based sensitivity estimates. Conclusions: Dialysis imposes substantial annual public expenditure in Greece. In this dataset and reimbursement context, peritoneal dialysis was more costly than hemodialysis, while private-sector hemodialysis generated lower recorded public-payer cost than public-sector provision. The apparent public–private hemodialysis cost difference was sensitive to the treatment of operating costs. These results may help inform reimbursement review, procurement policy and future budget-impact analyses of dialysis service redesign.

## 1. Introduction

Chronic kidney disease (CKD) is a progressive condition with major implications for patients, families and health systems. International guidelines define CKD using abnormalities of kidney structure or function that persist for at least three months and carry health consequences; recent global analyses show that CKD affects large populations and contributes substantially to morbidity and mortality worldwide [1,2,3,4]. When CKD progresses to end-stage renal disease (ESRD), renal replacement therapy (RRT) becomes necessary unless conservative care is selected. Dialysis, by hemodialysis (HD) or peritoneal dialysis (PD), is therefore both a life-sustaining intervention and a continuing fiscal commitment for health systems [5]. Resource requirements differ between acute and chronic RRT: acute RRT is typically an episodic hospital-based intervention for critically ill patients and may require continuous therapy and intensive monitoring, whereas chronic dialysis entails recurrent long-term treatments, dedicated infrastructure, staffing, transport and uninterrupted consumable supply [5].

Registry data emphasize the scale of this commitment. European and United States reports show large and persistent populations receiving RRT, with hemodialysis remaining the dominant treatment modality for most patients with ESRD [6,7,8]. In Greece, dialysis is universally available and is reimbursed through the National Organization for the Provision of Health Services (EOPYY) and public health budgets. This arrangement protects access, but it also places most dialysis expenditure directly on the public payer. The Greek dialysis population is highly dependent on in-center HD, while PD represents a smaller share of treatment. This modality distribution is relevant because the cost profile of dialysis depends not only on clinical need but also on reimbursement tariffs, consumable prices, staffing arrangements, patient transport, hospitalization and the allocation of unit operating costs.

The economic burden of CKD and ESRD extends beyond direct healthcare expenditure, including patient and family costs, productivity losses and informal care [9,10]. For public budget planning, however, direct public-payer expenditure remains the most actionable starting point. International studies show substantial heterogeneity in dialysis costs across countries and settings, with differences driven by labor prices, consumables, transport, dialysis frequency, home therapy use and reimbursement rules [11,12,13,14,15,16,17,18,19]. As a result, cost conclusions from one country cannot be transferred mechanically to another. Local micro-costing remains necessary when policy questions concern national reimbursement or service redesign.

Dialysis cost estimation is also analytically challenging because treatment costs are distributed across several budget lines. A narrow analysis of the dialysis session tariff can miss important public expenditure for drugs, hospitalizations, transport allowances and unit operation. Conversely, a broad societal analysis may be less useful for payers who need to know which costs are directly reimbursed and which are absorbed by provider budgets. For this reason, a transparent public-payer micro-costing approach can bridge clinical nephrology and health-policy decision-making by showing where expenditure is generated and which cost categories are most sensitive to local payment rules.

Greek evidence is informative but fragmented. Earlier studies estimated the cost of hemodialysis, the socioeconomic impact of dialysis, lifetime costs and QALYs across renal replacement options, and the relative cost of HD and PD in the years before and after the economic crisis [20,21,22,23,24]. A recent pediatric Greek analysis further illustrates that dialysis costs may differ by age group, modality mix and unit organization [25]. Nevertheless, there remains a need for updated adult patient-level estimates and a clear public-payer perspective. The objective of this study was to estimate the direct annual public-payer cost of dialysis therapies in Greece by modality, provider sector and treatment subtype, and to extrapolate these estimates to the national dialysis population.

## 2. Materials and Methods

### 2.1. Study Design, Setting and Perspective

We conducted a retrospective observational micro-costing study using calendar-year 2022 data. Reporting was structured according to STROBE principles for observational studies [26]. Cost identification, measurement and valuation followed standard health-economic costing principles [27]. The analytic perspective was the public payer and public health budget: costs were included when they were reimbursed by the public payer or borne by public hospitals. Costs borne exclusively by private providers, patients or families were outside the primary perspective.

Data were collected from five hemodialysis units and one peritoneal dialysis unit in Greece. The hemodialysis units comprised two public hospital units, two private hospital or clinic units and one private chronic hemodialysis center. The PD unit was located in a public hospital. This sampling structure allowed estimation of costs across modality and provider sector while retaining patient-level cost detail.

### 2.2. Study Population

Eligible patients were adults aged 18 years or older who received HD or PD continuously from 1 January to 31 December 2022 without treatment interruption, death, transplantation or modality change. Patients with incomplete or inconsistent data were excluded from the cost analysis. The final analytic sample included 359 patients: 337 receiving HD and 22 receiving PD.

### 2.3. Cost Categories and Valuation

For HD, treatment costs included statutory per-session reimbursement and the cost of dialyzers, blood lines and needles. Clawback adjustments (mandatory repayments to EOPYY when expenditure exceeds budget caps) were applied to private-sector reimbursement and material costs where relevant. For PD, treatment costs included PD fluids, bags, caps, automated PD components, catheter-related consumables and the fixed reimbursement for disposable medical supplies. These treatment costs were calculated at patient level from recorded utilization and unit prices.

Additional cost categories included allowances and transport reimbursement, dialysis-related medications, laboratory and imaging tests, hospitalization costs and dialysis-unit operating costs. Allowances included the nutritional allowance, air-therapy allowance and, for HD, transport reimbursement according to the relevant distance categories. Drug costs were based on executed prescriptions for dialysis-related medication categories. Laboratory and imaging costs were calculated from executed referrals. Hospitalization costs were calculated from recorded admissions and reimbursement categories. For public HD and PD units, the operating-cost variable was the aggregate annual patient-level allocation supplied from hospital accounting records. A component-level breakdown was not available; personnel, utilities, maintenance, depreciation and overhead were therefore represented only to the extent captured in the reported accounting allocation, and no additional component-specific imputation was undertaken. Corresponding private-sector operating costs were not added because they are financed by private providers within the reimbursed tariff and are not separately reimbursed by EOPYY.

To minimize double counting, each resource item was assigned to a single cost category. Costs already embedded in statutory dialysis reimbursement were not re-entered as separate patient-level items unless they were reimbursed separately, such as dialyzers and other dialysis materials. For public hospital units, the reported patient-level operating-cost allocation was added as a public-budget cost because it is borne by the hospital rather than by a private provider. For private units, EUR 0 was assigned to this category solely as a structural coding convention within the selected public-payer/public-budget perspective; it does not imply absence of personnel or other operating inputs. Consequently, public–private comparisons estimate differences in recorded public expenditure, not full economic production costs.

### 2.4. Statistical Analysis

Normality was assessed using the Shapiro–Wilk and Kolmogorov–Smirnov tests. Continuous variables were summarized using means and standard deviations or medians and interquartile ranges (IQRs), as appropriate. Because cost data were non-normally distributed and right-skewed, they were summarized using medians and IQRs. Means and standard deviations were reported only where they were useful for budgetary extrapolation or comparison with prior cost studies. The primary statistical analysis used unweighted patient-level data, with each patient contributing one observation. Differences between two independent groups were assessed using Mann–Whitney U tests and differences across more than two groups were assessed using Kruskal–Wallis tests. Statistical significance was assessed at *p* < 0.05. To assess whether asymmetric visibility of operating costs affected the main findings, sensitivity analyses recalculated total costs after excluding dialysis-unit operating costs from all HD and PD observations and repeated the HD-versus-PD and private-versus-public HD comparisons. Statistical analyses were performed using SPSS, Version 24.0.

For national expenditure estimation, subtype-specific mean and median costs were applied to prevalent national modality counts on 31 December 2022 for conventional HD, hemodiafiltration (HDF), continuous ambulatory peritoneal dialysis (CAPD) and automated peritoneal dialysis (APD), as reported by the ERA Registry Annual Report 2022 [7]. The registry reported 7990 center-based HD patients, 1 home-HD patient, 3958 HDF patients, 220 CAPD patients and 402 APD patients. Because a separate home-HD cost estimate was unavailable, the single home-HD patient was grouped with conventional HD; this had an immaterial effect on the national total. For budgetary extrapolation, mean-based estimates were used as the primary analysis because aggregate expenditure is mathematically determined by the mean cost per patient multiplied by the number of patients. Median-based estimates were additionally reported as a conservative sensitivity analysis, given the right-skewed distribution of individual patient costs.

## 3. Results

### 3.1. Sample Characteristics

The final sample included 359 patients (337 HD and 22 PD). Among HD patients, 253 (75.1%) were treated in private-sector units and 84 (24.9%) in public-sector units. Demographic and treatment-subtype characteristics are summarized in Table 1.

### 3.2. Annual Costs by Modality and Provider Sector

Cost structures differed by modality: HD costs were distributed across sessions/materials, allowances/transport and medication, whereas PD costs were concentrated in consumables, particularly APD supplies (Table 2). Median annual total cost was EUR 35,902.53 for HD and EUR 57,136.44 for PD. The modality difference was driven mainly by higher PD treatment and consumable costs; HD had higher allowance/transport and medication costs. Hospitalization and dialysis-unit operating costs had highly skewed distributions.

Among HD patients, recorded median annual public-payer/public-budget cost was higher in public than private units (EUR 38,794.55 versus EUR 34,836.98; Table 3). This primary comparison includes public-unit operating-cost allocations but no corresponding private-provider operating costs and therefore should not be interpreted as a full production-cost or efficiency comparison.

### 3.3. Treatment Subgroups

Treatment subtype was a major cost driver. Conventional HD had a median annual total cost of EUR 31,531.43, whereas HDF had a median of EUR 39,158.29. The difference was expected because HDF carries higher reimbursed session and consumable costs. Within PD, CAPD had a median annual total cost of EUR 47,737.95, whereas APD had a median of EUR 73,055.91, again reflecting the higher cost of automated treatment components and supply intensity (Table 3).

### 3.4. Inferential Cost Comparisons

Non-parametric comparisons supported the descriptive findings (Table 4). Costs differed across the five HD units; private-sector HD had lower recorded treatment and total costs but higher laboratory/imaging and medication costs and lower hospitalization costs than public-sector HD.

HDF cost more than conventional HD, and APD cost more than CAPD (all *p* < 0.001; Table 4). PD also had a higher total annual cost than HD (*p* < 0.001). In the sensitivity analysis excluding dialysis-unit operating costs from every observation, median annual cost remained higher for PD than HD (EUR 53,410.71 versus EUR 35,227.63; *p* < 0.001). The private–public HD difference attenuated to EUR 34,836.98 versus EUR 36,731.57 and was no longer statistically significant (*p* = 0.064), confirming that the primary sector difference was largely driven by asymmetric visibility of operating costs.

### 3.5. Estimated National Public Expenditure

Using 2022 national modality counts, mean-based annual public expenditure was estimated at EUR 427 million for HD and EUR 39.5 million for PD, totaling EUR 466.6 million. The median-based sensitivity estimate was EUR 407 million for HD and EUR 39.9 million for PD, totaling EUR 446.8 million (Table 5). The difference between mean- and median-based totals mainly reflects right-skewness in HD costs.

## 4. Discussion

This patient-level micro-costing study shows that dialysis generates substantial annual public expenditure in Greece. Median annual total cost was EUR 35,902.53 for HD and EUR 57,136.44 for PD. Using 2022 national modality counts, estimated annual public expenditure ranged from EUR 446.8 million in the median-based sensitivity analysis to EUR 466.6 million in the mean-based base case. These figures place dialysis among the continuing high-cost obligations of the Greek health system and provide updated inputs for reimbursement and budget-impact analyses.

A central finding is that PD was more costly than in-center HD in the Greek public-payer/public-budget context. This contrasts with many international studies in which PD is often less costly or more cost-effective than HD, especially in systems where in-center HD labor, infrastructure and transport costs are high [11,12,13,14,15,16,28]. It is also consistent with the broader literature showing that dialysis economics are highly context-dependent and strongly influenced by reimbursement rules, provider organization and consumable prices [17,18,19]. In this Greek dataset, PD cost was dominated by materials and consumables, particularly for APD. Importantly, PD remained more costly than HD after unit operating costs were excluded from both modalities, indicating that the finding was not produced solely by asymmetric accounting of private-sector HD operating costs. Nevertheless, this is a payer-specific result reflecting current Greek procurement and reimbursement rather than a full comparison of economic production costs. The policy implication is therefore not that PD is clinically or economically unattractive in general, but that its current procurement and reimbursement profile in Greece may limit its potential as a cost-containment strategy.

The provider-sector comparison requires even greater caution. Private-sector HD had lower recorded public-payer cost than public-sector HD in the primary analysis because public hospitals bear and report dialysis-unit operating costs, whereas private providers finance operating inputs within the reimbursed tariff and these costs do not appear as a separate public-budget line. Operating-cost data were available only as aggregate patient-level allocations from public hospital accounting, without a component-level breakdown of personnel, utilities, maintenance, depreciation or overhead; corresponding private-sector data were unavailable. When operating costs were excluded from all HD observations, the sector difference was no longer statistically significant. The primary comparison therefore reflects budget visibility rather than demonstrated productive efficiency. A full economic costing study would need comparable private- and public-sector data on staffing, capital, maintenance, depreciation and overhead, as well as patient and family costs.

The higher cost of HDF relative to conventional HD is universally acknowledged and was shown to be statistically robust. HDF may offer clinical advantages for selected patients, but it also carries higher session and consumable costs. The present study was not designed to assess cost-effectiveness, survival or quality of life, and should therefore not be interpreted as evidence against widespread HDF use. Instead, the results quantify the budget impact of the existing HDF share and provide a basis for future analyses that combine costs with outcomes.

The results are broadly comparable with earlier Greek studies, although direct comparison is difficult because prior work used different years, perspectives, cost categories and patient populations [20,21,22,23,24]. The finding that dialysis remains expensive is consistent across the Greek literature. The present analysis adds updated 2022 data and 2022 national modality counts, separates provider sector, distinguishes conventional HD from HDF and CAPD from APD, and includes formal inferential comparisons from patient-level data. The recent Greek pediatric cost analysis further reinforces that modality-specific costs in Greece can differ materially from assumptions derived from adult or international populations [25].

The generalizability of the numerical estimates to other healthcare systems is limited. The micro-costing framework and resource categories may be transferable, but absolute costs and even the relative ranking of modalities will depend on local tariffs, provider mix, labor and consumable prices, transport coverage, home-dialysis uptake and whether operating costs are separately visible to the payer [17,18,19]. The estimates should therefore be used as a context-specific Greek benchmark rather than directly extrapolated to other healthcare systems.

The findings have several policy implications. First, the design of policies targeting the expansion of PD use should include a careful review of consumable procurement, given that PD treatment costs dominated total PD expenditure. Second, reimbursement policy should avoid interpreting lower private-sector recorded expenditure as proof of lower full economic cost, particularly because the sector difference attenuated when public operating-cost allocations were excluded. Third, national planning should account for the cost consequences of the current HDF share and the growing need for dialysis in an ageing population. Finally, these results can serve as the empirical cost base for a separate budget-impact or investment-appraisal study of home hemodialysis and other service redesign options.

From a health-services perspective, the study also highlights why modality policy should not be based on cost alone. PD and HD differ in patient eligibility, autonomy, travel burden, home support requirements, complications and patient preferences. A reimbursement review aimed at reducing PD costs could enhance the economic attractiveness of home-based therapy, but implementation would still depend on patient education, catheter services, training capacity and clinical follow-up. Similarly, any policy to expand or limit HDF should consider both budget impact and clinical criteria. The present results therefore provide a cost foundation rather than a complete decision rule.

## 5. Limitations

This study has limitations. First, data were obtained from five HD units and one PD unit, and the sample may not capture all regional variation in Greece. However, the main drivers of total cost—reimbursement tariffs, consumable prices and allowances—are largely standardized nationally. Second, the PD sample was small, particularly for CAPD–APD comparison; these estimates should therefore be confirmed in a larger multicenter sample. Third, the retrospective analysis depended on the completeness and accuracy of medical, prescription, referral and accounting records.

Fourth, the perspective was deliberately restricted to the public payer and public budget. This is appropriate for reimbursement and fiscal planning, but it excludes private provider operating costs, patient and caregiver time, productivity losses and informal care. The resulting asymmetric visibility of operating costs may overstate sector differences in full economic cost; indeed, the public–private HD difference was no longer statistically significant after operating costs were excluded. Public operating costs were also available only as aggregate patient-level allocations, precluding component-level validation. Fifth, the study estimated costs but not outcomes and cannot determine whether one modality is clinically preferable or cost-effective. Sixth, national extrapolations used year-end 2022 registry counts as a proxy for the annual treated population and assumed that sample-derived subtype costs were nationally representative; patient turnover, regional case mix or changes in modality share could affect the estimates.

Finally, the estimates reflect 2022 prices, reimbursement rules and practice patterns. Future changes in clawback rates, dialysis tariffs, drug prices, transport policy, public hospital accounting or consumable contracts could materially alter the cost profile. Periodic updating with administrative data would therefore be valuable, particularly if Greece reforms dialysis reimbursement or introduces new home-dialysis policies.

## 6. Conclusions

Dialysis care imposes a high and continuing public expenditure burden in Greece. In this public-payer/public-budget analysis, PD remained more costly than HD even after dialysis-unit operating costs were excluded. Public-sector HD had higher recorded cost in the primary analysis, but the sector difference was not statistically significant after removing operating costs and should not be interpreted as evidence of higher full production cost. These findings support evidence-informed reimbursement review, procurement planning and future economic evaluations of dialysis service redesign in Greece.

## Figures and Tables

**Table 1:** Selected sample characteristics.

Characteristic	Overall	Hemodialysis	Peritoneal Dialysis
Patients, n	359	337	22
Male sex, n (%)	250 (69.6)	238 (70.6)	12 (54.5)
Median age, years (range)	70 (24–95)	70 (24–95)	64.5 (25–95)
Private-sector treatment, n (%)	253 (70.5)	253 (75.1)	0 (0.0)
Public-sector treatment, n (%)	106 (29.5)	84 (24.9)	22 (100)
Treatment subtype, n (%)	Conventional HD: 199 (55.4); HDF: 138 (38.4); CAPD: 10 (2.8); APD: 12 (3.3)	Conventional HD: 199 (59.1); HDF: 138 (40.9)	CAPD: 10 (45.5); APD: 12 (54.5)

HD, hemodialysis; HDF, hemodiafiltration; CAPD, continuous ambulatory peritoneal dialysis; APD, automated peritoneal dialysis.

**Table 2:** Median annual patient-level public-payer cost by modality and cost category.

Cost Category	Hemodialysis, Median (IQR), EUR	Peritoneal Dialysis, Median (IQR), EUR
Treatment/dialysis consumables	19,328.40 (18,179.72–25,095.59)	46,510.50 (36,525.25–62,131.75)
Allowances and transport reimbursement	7304.00 (7304.00–7304.00)	4544.00 (4544.00–4544.00)
Dialysis-related medications	3203.03 (2035.73–4303.10)	792.21 (545.99–1741.71)
Laboratory and imaging tests	360.69 (134.72–805.13)	791.19 (550.48–891.56)
Hospitalizations	0.00 (0.00–2248.10)	1061.10 (1061.10–1061.10)
Dialysis-unit operating cost	0.00 (0.00–501.54)	3725.73 (3725.73–3725.73)
Total annual cost	35,902.53 (30,963.14–40,180.19)	57,136.44 (47,989.21–74,585.62)

IQR, interquartile range. Component medians are not expected to sum exactly to the median total cost. Zero median values do not indicate absence of cost for every patient. Hospitalization cost was EUR 0 for 171 of 337 HD patients and positive for 166, producing a zero median with a positive upper quartile. Dialysis-unit operating cost was positive for the 84 public-sector HD patients and structurally coded as EUR 0 for the 253 private-sector HD patients because private operating costs were outside the selected public-payer/public-budget perspective. The pooled HD operating-cost median was therefore EUR 0, with the upper quartile lying at the boundary between structural zeros and positive public-unit allocations. These values should not be interpreted as complete provider production costs.

**Table 3:** Median annual cost by treatment subgroup.

Treatment Subgroup	n	Total Annual Cost, Median (IQR), EUR	Treatment Cost, Median (IQR), EUR
Conventional HD	199	31,531.43 (28,753.68–35,213.66)	19,204.57 (17,447.43–19,328.40)
HDF	138	39,158.29 (37,248.93–43,823.55)	27,836.39 (25,095.59–27,836.39)
Public-sector HD	84	38,794.55 (34,553.02–42,660.40)	19,432.30 (19,328.40–27,996.70)
Private-sector HD	253	34,836.98 (29,453.65–38,806.62)	19,304.10 (17,447.43–25,095.59)
CAPD	10	47,737.95 (46,794.32–49,597.81)	36,478.50 (35,484.00–37,800.00)
APD	12	73,055.91 (62,420.18–79,658.17)	61,524.00 (51,071.15–67,741.00)

HD, hemodialysis; HDF, hemodiafiltration; CAPD, continuous ambulatory peritoneal dialysis; APD, automated peritoneal dialysis.

**Table 4:** Summary of principal inferential cost comparisons.

Comparison	Cost Outcome	Test Statistic	*p* Value
Five HD units	Treatment cost	Kruskal–Wallis H(4) =63.08	<0.001
Five HD units	Total annual HD cost	Kruskal–Wallis H(4) = 60.69	<0.001
Private vs. public HD	Treatment cost	U = 5809; z = −6.26	<0.001
Private vs. public HD	Total annual HD cost	U = 6713; z = −5.06	<0.001
Private vs. public HD	Laboratory/imaging cost	U = 16,275.5; z = 7.31	<0.001
Private vs. public HD	Medication cost	U = 12,705; z = 2.69	0.007
Private vs. public HD	Hospitalization cost	U = 8472; z = −2.99	0.003
Conventional HD vs. HDF	Treatment cost	U = 27,462; z = −15.70	<0.001
Conventional HD vs. HDF	Total annual HD cost	U = 23,783; z = −11.43	<0.001
HD vs. PD	Total annual cost	U = 7110; z = −7.22	<0.001
CAPD vs. APD	PD treatment cost	U = 118; z = −3.82	<0.001
CAPD vs. APD	Total annual PD cost	U = 117; z = −3.76	<0.001

All tests use unweighted patient-level data. Negative z values indicate that the first-named group has lower costs than the second-named group.

**Table 5:** Estimated national annual public expenditure for dialysis in Greece.

Dialysis Modality	National Patient Count, n	Mean-Based Annual Cost per Patient, EUR	Median-Based Annual Cost per Patient, EUR	Mean-Based National Expenditure Estimate, EUR	Median-Based National Expenditure Sensitivity Estimate, EUR
Conventional HD	7991	33,085.43	31,531.43	264,385,644	251,967,651
HDF	3958	41,093.26	39,158.29	162,647,105	154,988,528
All HD	11,949	35,737.95	34,057.76	427,032,749	406,956,178
CAPD	220	47,688.38	47,737.95	10,491,444	10,502,348
APD	402	72,241.47	73,055.91	29,041,072	29,368,477
All PD	622	63,557.10	64,101	39,532,516	39,870,825
All dialysis	12,571	37,114.41	35,544.27	466,565,265	446,827,003

National expenditure estimates were obtained by applying modality-specific annual costs to 2022 ERA prevalence counts for HD, HDF, CAPD and APD [7]; the single home-HD patient was grouped with conventional HD because a separate cost estimate was unavailable. For individual modalities, per-patient values are modality-specific estimates. For All HD, All PD and All dialysis, per-patient values are population-weighted averages across subtypes, with weights based on national subtype counts. Base-case expenditure uses mean patient-level cost because total expenditure is arithmetically mean-driven; the median-based estimate is provided as a conservative sensitivity analysis. The All dialysis median-based value is a weighted average of modality-specific medians, not a pooled national patient-level median. National expenditure estimates were calculated using unrounded per-patient cost estimates; per-patient values presented in the table are rounded to two decimal places. Consequently, minor differences may arise when displayed values are multiplied directly.

## Data Availability

Dataset available on request from the authors.

## Abbreviations

The following abbreviations are used in this manuscript:

APD	Automated peritoneal dialysis
CAPD	Continuous ambulatory peritoneal dialysis
CKD	Chronic kidney disease
EOPYY	National Organization for the Provision of Health Services
ESRD	End-stage renal disease
HD	Hemodialysis
HDF	Hemodiafiltration
IQR	Interquartile range
PD	Peritoneal dialysis
RRT	Renal replacement therapy

## References

[1] (2024). Kidney Disease: Improving Global Outcomes (KDIGO) CKD Work Group. KDIGO 2024 clinical practice guideline for the evaluation and management of chronic kidney disease. Kidney Int..

[2] GBD Chronic Kidney Disease Collaboration (2020). Global, regional, and national burden of chronic kidney disease, 1990–2017: A systematic analysis for the Global Burden of Disease Study 2017. Lancet.

[3] Jha V., Garcia-Garcia G., Iseki K., Li Z., Naicker S., Plattner B., Saran R., Wang A.Y.-M., Yang C.-W. (2013). Chronic kidney disease: Global dimension and perspectives. Lancet.

[4] Bello A.K., Levin A., Lunney M., A Osman M.A., Ye F., E Ashuntantang G.E., Bellorin-Font E., Gharbi M.B., Davison S.N., Ghnaimat M. (2019). Status of care for end stage kidney disease in countries and regions worldwide: International cross-sectional survey. BMJ.

[5] Himmelfarb J., Vanholder R., Mehrotra R., Tonelli M. (2020). The current and future landscape of dialysis. Nat. Rev. Nephrol..

[6] Boenink R., Bonthuis M., Boerstra B.A., Astley M.E., de Sousa I.R.M., Helve J., Komissarov K.S., Comas J., Radunovic D., Buchwinkler L. (2024). The ERA Registry Annual Report 2022: Epidemiology of kidney replacement therapy in Europe, with a focus on sex comparisons. Clin. Kidney J..

[7] ERA Registry (2024). ERA Registry Annual Report 2022.

[8] United States Renal Data System (2024). 2024 USRDS Annual Data Report: Epidemiology of Kidney Disease in the United States.

[9] Wang V., Vilme H., Maciejewski M.L., Boulware L.E. (2016). The economic burden of chronic kidney disease and end-stage renal disease. Semin. Nephrol..

[10] de Vries E.F., Los J., de Wit G.A., Hakkaart-van Roijen L. (2021). Patient, family and productivity costs of end-stage renal disease in the Netherlands; Exposing non-healthcare-related costs. BMC Nephrol..

[11] Karopadi A.N., Mason G., Rettore E., Ronco C. (2013). Cost of peritoneal dialysis and haemodialysis across the world. Nephrol. Dial. Transplant..

[12] Klarenbach S.W., Tonelli M., Chui B., Manns B.J. (2014). Economic evaluation of dialysis therapies. Nat. Rev. Nephrol..

[13] Roberts G., Holmes J., Williams G., Chess J., Hartfiel N., Charles J.M., McLauglin L., Noyes J., Edwards R.T. (2022). Current costs of dialysis modalities: A comprehensive analysis within the United Kingdom. Perit. Dial. Int..

[14] Mushi L., Marschall P., Fleßa S. (2015). The cost of dialysis in low- and middle-income countries: A systematic review. BMC Health Serv. Res..

[15] Mohnen S.M., van Oosten M.J.M., Los J., Leegte M.J.H., Jager K.J., Hemmelder M.H., Logtenberg S.J.J., Stel V.S., Roijen L.H.-V., de Wit G.A. (2019). Healthcare costs of patients on different renal replacement modalities: Analysis of Dutch health insurance claims data. PLoS ONE.

[16] Baboolal K., McEwan P., Sondhi S., Spiewanowski P., Wechowski J., Wilson K. (2008). The cost of renal dialysis in a UK setting—A multicentre study. Nephrol. Dial. Transplant..

[17] Icks A., Haastert B., Gandjour A., Chernyak N., Rathmann W., Giani G., Rump L.-C., Trapp R., Koch M. (2010). Costs of dialysis—A regional population-based analysis. Nephrol. Dial. Transplant..

[18] Vanholder R., Davenport A., Hannedouche T., Kooman J., Kribben A., Lameire N., Lonnemann G., Magner P., Mendelssohn D., Saggi S.J. (2012). Reimbursement of dialysis: A comparison of seven countries. J. Am. Soc. Nephrol..

[19] Vanholder R., Annemans L., Brown E., Gansevoort R., Gout-Zwart J.J., Lameire N., Morton R.L., Oberbauer R., Postma M.J., Tonelli M. (2017). Reducing the costs of chronic kidney disease while delivering quality health care: A call to action. Nat. Rev. Nephrol..

[20] Kaitelidou D., Ziroyanis P.N., Maniadakis N., Liaropoulos L.L. (2005). Economic evaluation of hemodialysis: Implications for technology assessment in Greece. Int. J. Technol. Assess. Health Care.

[21] Kaitelidou D., Ziroyiannis P.N., Maniadakis N., Liaropoulos L., Theodorou M. (2004). The socioeconomic impact of hemodialysis. Hippokratia.

[22] Kontodimopoulos N., Niakas D. (2008). An estimate of lifelong costs and QALYs in renal replacement therapy based on patients’ life expectancy. Health Policy.

[23] Koukou M.G., Smyrniotis V.E., Arkadopoulos N.F., Grapsa E.I. (2017). PD vs. HD in post-economic crisis Greece—Differences in patient characteristics and estimation of therapy cost. Perit. Dial. Int..

[24] Naoum P., Topkaroglou I., Kitsonis D., Skroumpelos A., Athanasakis K., Iatrou C., Boletis J., Kyriopoulos J. (2016). Cost calculations during “dire straits”: A cost-of-illness analysis of regular hemodialysis for end-stage renal disease in Greece. Int. J. Artif. Organs.

[25] Ntais C., Loizou K., Panagiotakis C., Kontodimopoulos N., Fanourgiakis J. (2024). Cost analysis of end-stage renal disease in pediatric patients in Greece. Healthcare.

[26] von Elm E., Altman D.G., Egger M., Pocock S.J., Gotzsche P.C., Vandenbroucke J.P., STROBE Initiative (2007). The Strengthening the Reporting of Observational Studies in Epidemiology (STROBE) Statement: Guidelines for reporting observational studies. PLoS Med..

[27] Drummond M.F., Sculpher M.J., Claxton K., Stoddart G.L., Torrance G.W. (2015). Methods for the Economic Evaluation of Health Care Programmes.

[28] Klomjit N., Kattah A.G., Cheungpasitporn W. (2020). The cost-effectiveness of peritoneal dialysis is superior to hemodialysis: Updated evidence from a more precise model. Kidney Med..

